# Multiple Tumor Suppressor microRNAs Regulate Telomerase and TCF7, an Important Transcriptional Regulator of the Wnt Pathway

**DOI:** 10.1371/journal.pone.0086990

**Published:** 2014-02-14

**Authors:** Radmila Hrdličková, Jiří Nehyba, William Bargmann, Henry R. Bose

**Affiliations:** Department of Molecular Biosciences, College of Natural Sciences, and Institute for Cellular and Molecular Biology, The University of Texas at Austin, Austin, Texas, United States of America; IPMC, CNRS UMR 7275 UNS, France

## Abstract

The human TERT (hTERT) gene encodes the telomerase catalytic subunit which plays a role in telomerase regulation. Telomerase is activated in more than 90% of all human malignancies and understanding how telomerase is regulated is necessary for implementation of successful anti-cancer therapies. microRNAs (miRNAs) are important regulators of gene expression in eukaryotic cells but evidence of their role in telomerase regulation has not been documented. To determine whether hTERT activity is regulated by multiple miRNAs, eight miRNAs which have putative binding sites in the hTERT 3′UTR together with miR-138-5p were evaluated in luciferase assays with a reporter containing the hTERT 3′UTR. Six miRNAs (let-7g*, miR-133a, miR-138-5p, miR-342-5p, miR-491-5p, and miR-541-3p) specifically inhibited the expression of the reporter luciferase-driven constructs and let-7g*, miR-133a, miR-138-5p, and miR-491-5p also downregulated endogenous telomerase activity in cells. Moreover, all six miRNAs significantly inhibited cell proliferation. miRNAs (miR-133a, miR-138-5p, 342-5p, 491-5p, 541-3p) also have predicted binding sites within the 3′UTR of three genes involved in Wnt signaling (TCF7, MSI1, and PAX5). These miRNAs inhibited the expression of the luciferase reporter constructs containing 3′UTRs of these genes and downregulated protein expression of the TCF7 transcription factor, which mediates the canonical Wnt pathway. Together, these results suggest the existence of a miRNA regulatory network involving the hTERT and Wnt pathway.

## Introduction

MicroRNAs (miRNAs) are a class of short non-protein-coding RNA molecules that bind with Argonaute proteins to form RNA-induced silencing complexes (RISCs) [1]. These complexes modulate the expression of other genes by translational repression and mRNA degradation [2], [3]. miRNAs provide the target specificity to the RISC by base-pair interactions with the targeted mRNA molecules [4]. In animal species, miRNA binding sites in mRNA molecules are usually concentrated in the 3′ untranslated region (3′UTR) [5]. The primary miRNA transcripts form hairpin structures that are processed by nuclear and cytoplasmic enzymatic complexes to yield a mature form [6], [7]. Each hairpin can potentially encode two mature targeting miRNAs that are designated as 5p or 3p indicating that they are processed respectively from the 5′ or 3′ arm of the hairpin. One of the mature miRNAs usually accumulates in the cell at lower levels and is often designated as the miRNA* species. The number of miRNA genes in Metazoa increases with the structural complexity of metazoan species from approximately ten in the basal branch of Porifera (sea sponges) to over one thousand in vertebrates [8]. This correlation suggests an important role for miRNAs in the evolution of complexity in animal species [9].

miRNAs are key regulators of embryogenesis and establishment of tissue identity in vertebrates [10], [11]. In undifferentiated embryonic cells, miRNAs participate in clearing maternal transcripts and repress negative regulators of proliferation. In differentiated cells, different sets of miRNAs silence pluripotency regulators and promote differentiation. Because of this dual role, miRNAs participate in distinct aspects of cancer progression [12], [13]. Individual miRNAs have been found to act either as oncogenes or anti-oncogenes [14]. Various miRNAs were shown to be useful markers for different cancers and are promising targets for cancer therapy [15], [16]. To understand the role of miRNAs in the multistep process of cancer progression it is necessary to determine how they regulate genes critical for tumorigenesis.

Replicative immortality, one of the hallmarks of cancer progression, is dependent on the activation of telomerase in most human tumors [17]–[19]. Telomeric extension is carried out by a multisubunit complex, which consists of the telomerase reverse transcriptase (TERT), the telomerase RNA component, as well as other associated proteins [20]. By adding telomeric repeats (TTAGGG) to the ends of chromosomes, telomerase protects telomeres from erosion caused by the inability of the DNA replication apparatus to duplicate extreme 5′ ends of linear DNA and also by oxidative damage [21], [22]. Other telomerase activities include the stimulation of cell proliferation, protection against oxidative damage and apoptosis, modulation of global gene expression, activation of stem cells, and tumor promotion [23], [24]. Human TERT (hTERT) interacts with the chromatin remodeling factor, BRG1, and as a component of a TCF/β-catenin transcription complex, binds to promoters of Wnt target genes and activates their transcription [25].

Telomerase expression and activity in vertebrates is tightly controlled. Telomerase is active in embryonic tissues but downregulated in most adult somatic cells [26]. Telomerase activity is regulated through several of its components and interacting molecules [27]. The expression of hTERT is primarily determined by the transcriptional activity of the hTERT gene promoter and post-transcriptional modifications of the hTERT mRNA by alternative splicing [28]–[30]. Non-coding RNAs have also been implicated in the regulation of hTERT expression. miR-138-5p was shown to regulate hTERT in thyroid carcinoma cells by directly interacting with hTERT mRNA [31].

In this report we demonstrate that hTERT is regulated by multiple miRNAs and that this regulatory network is interconnected with other pathways that are also involved in oncogenesis.

## Results

### Identification of miRNAs

**Figure 1:**
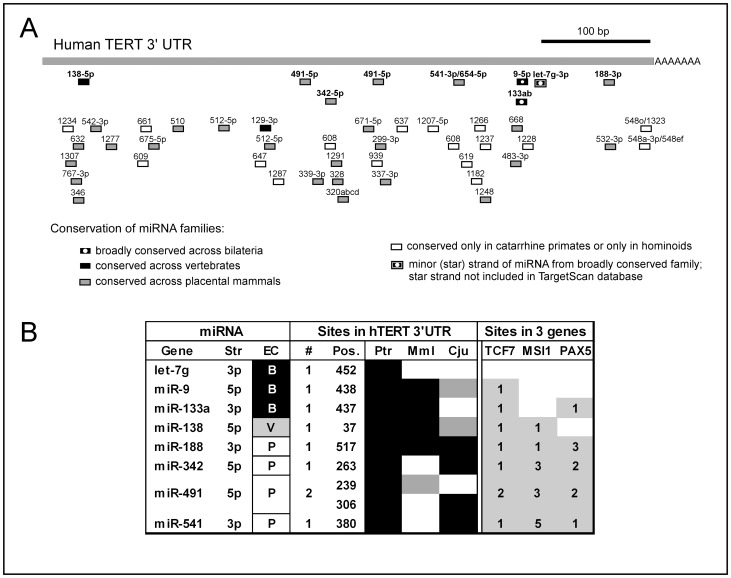
Selection of miRNA target sites in the 3′UTR of human TERT. (a) miRNA target sites predicted by TargetScanHuman 5.2 (http://www.targetscan.org/). The horizontal bar on the right upper corner represents 100 kb scale. (b) miRNAs selected for experimental evaluation. The table identifies the miRNA gene, hairpin strand encoding the mature targeting strand (Str), the evolutionary conservation (EC) of the specific miRNA family: B - conserved in Bilateria, V - across vertebrates, P - in placental mammals. Number of targeting sites in the hTETR 3′UTR is indicated (#) together with the position in the 3′UTR (Pos). Evolutionary conservation of the sites is shown in the chimpanzee (Ptr), rhesus macaque (Mml), and common marmoset (Cja) indicated by black boxes (absolute conservation) or gray boxes (conserved when wobble G-U pairing is considered). Presence of the sites in the 3′UTR of the three genes of the Wnt pathway is indicated by a number in the gray field.

The miR-138-5p was previously shown to bind a site in the hTERT 3′UTR [31]. Further, a conference report suggested that let-7g-3p (called let-7g*) also interacts with the hTERT mRNA in pulmonary fibrosis cells [32]. Many genes involved in cancer contain mRNA 3′UTRs that are targeted by multiple miRNAs and these regulatory networks act to control genes that contribute to carcinogenesis [33]. We hypothesized that if hTERT is involved in a gene-miRNA regulatory network, then functional miRNA binding sites located in hTERT mRNA will also be clustered together in the 3′UTRs of other genes that belong to the same network. We have used the TargetScan prediction tool to identify miRNAs which share predicted binding sites in the 3′UTR of hTERT and other genes [34]. TargetScanHuman predicted 47 miRNA binding sites in the hTERT 3′UTR (Figure 1a). We used TargetScan to select miRNAs which also likely regulate other genes (Methods S1). We selected four miRNAs (miR-188-3p, 342-5p, 491-5p, and 541-3p), which have binding sites in the 3′UTR of three other genes, TCF7, MSI1, and PAX5 (Figure 1b). These genes are involved in Wnt pathway [35]–[39]. Additional miRNAs for further analysis were selected based on their broad conservation across most bilaterian metazoans (miR-9-5p, miR-133a) or because they have previously been shown to interact with the hTERT 3′UTR (miR-138-5p, let-7g*) [31], [32]. miR-138-5p and -133a also have potential binding sites in two out of the three selected genes, while a miR-9 is predicted to have two sites in TCF7 (Figure 1b). All of the binding sites for the eight selected miRNAs are conserved in the chimpanzee (Ptr) TERT 3′UTR, while only some of these miRNA binding sites are present in macaque (Mml) and the common marmoset (Cja) TERT.

The TCF7 3′UTR has similar numbers of predicted binding sites for seven different miRNAs found in the hTERT 3′UTR, lacking only binding sites for let-7g* (Figure 1b). The other two genes, MSI1 and PAX5, share binding sites for five different miRNAs, including multiple sites for miR-188-3p, 342-5p, 491-5p and 541-3p (twelve in MSI1 and eight in PAX5 3′UTRs) with the 3′UTR of hTERT. In conclusion, we selected eight miRNAs with predicted binding sites in hTERT 3′UTR of which the majority also share binding sites with three genes involved in Wnt pathway for further analysis.

### Luciferase Reporter Screens Indicate that Multiple miRNAs Target the hTERT 3′UTR

**Figure 2:**
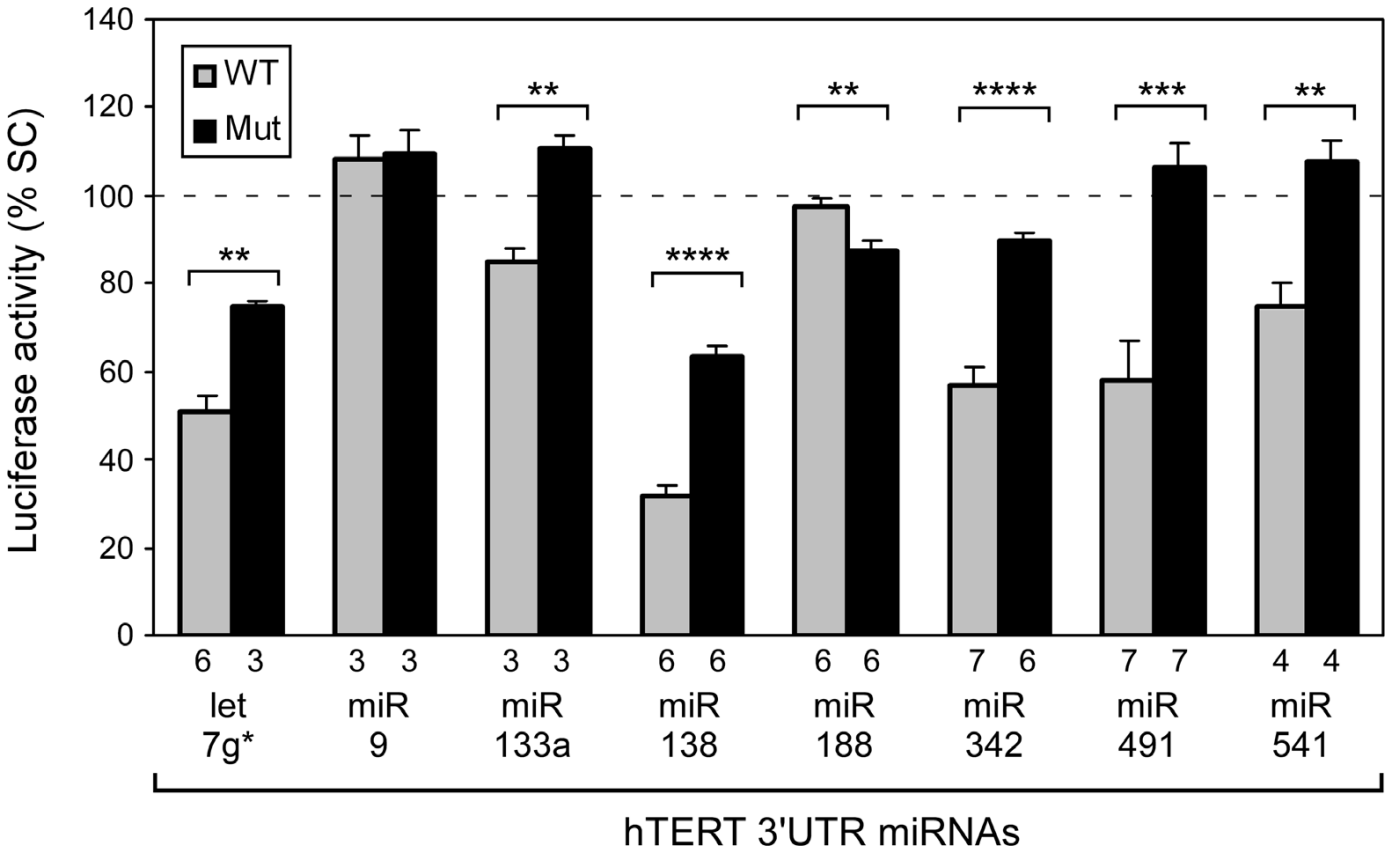
Several miRNAs target the hTERT 3′UTR. Downregulation of luciferase activity of the hTERT 3′UTR reporter in HeLa cells transfected with miRNA mimic molecules (Table S1). The results are expressed as percent of reporter activity in cells transfected with a scrambled control (SC). The specificity of miRNA effect was evaluated by comparing the luciferase activity of wild type hTERT 3′UTR reporter (WT, gray bars) with a set of mutant 3′UTR reporters (Mut, black bars) in HeLa cells transfected with miRNA mimics. The reporter activity of the mutated construct in cells transfected with miRNA mimics is expressed as percent of mutated reporter activity in parallel cell cultures transfected with a scrambled control. Means and standard errors were calculated from 3–7 values (numbers are below bars) from 4 independent experiments. Statistically significant differences relative to wild type 3′UTR are indicated. *P* values for differences were determined by two-tailed Student′s *t* test (*P<0.05, **P<0.01, ***P<0.001, **** *P<*0.0001).

To test if any of the selected miRNAs could regulate hTERT gene expression through its predicted binding sites in the hTERT 3′UTR, we used a luciferase reporter assay (Figure 2). HeLa cells were co-transfected with a vector containing the hTERT 3′UTR (WT) downstream of the luciferase gene and with miRNA precursor molecules which mimic endogenous miRNAs. The precursors of scrambled miRNA were used as negative controls. To test the binding specificity, parallel assays were performed with reporter constructs expressing the 3′UTRs with relevant miRNA sites altered by site-directed mutagenesis (Mut). The differences in luciferase activity between cells treated with the transfection reagents only and the negative miRNA control were not statistically significant (data not shown). The luciferase activity of the WT reporter construct was significantly inhibited in cells transfected with precursors of let-7g*, miR-133a, miR-138, miR-342, miR-491, and miR-541 relative to cells transfected with the negative control. The inhibitory effect observed for miR-133a, miR-342, miR-491 and miR-541, was completely or almost completely eliminated when the luciferase assays employed the hTERT 3′UTR constructs with mutated binding sites. The inhibitory effects of miR-138 and let-7g* were greatly reduced for mutated reporters. Residual activity of the mutated reporter constructs with these two miRNAs can be explained by the presence of other, undetected binding site(s) in the 3′UTR of hTERT, failure to completely eliminate binding by mutagenesis, or that part of the inhibitory effect is not mediated through the binding of miRNAs to the hTERT 3′UTR. Nevertheless, mutagenesis significantly reduced the inhibition of the reporter constructs by these two miRNAs demonstrating that most of their effect is mediated through the predicted binding sites. The miR-9, and miR-188 had no significant inhibitory effect on the WT reporter activity relative to the scrambled miR control. Mutation of miR-9 binding site did not change the activity of the reporter in miR-9 transfected cells while mutation of the miR-188 binding site slightly increased the susceptibility of the reporter to the inhibition by the miR mimic. Preliminary immunoprecipitation of Argonaute complexes with miR-138 supported the luciferase results suggesting that miRNAs effect is due to binding predicted miRNA sites (data not shown). These assays demonstrated that let-7g*, miR-133a, miR-138, miR-342, miR-491, and miR-541 were able to functionally interact with the 3′UTR of hTERT via their predicted binding sites.

### miRNAs Modulate Telomerase Activity

**Figure 3:**
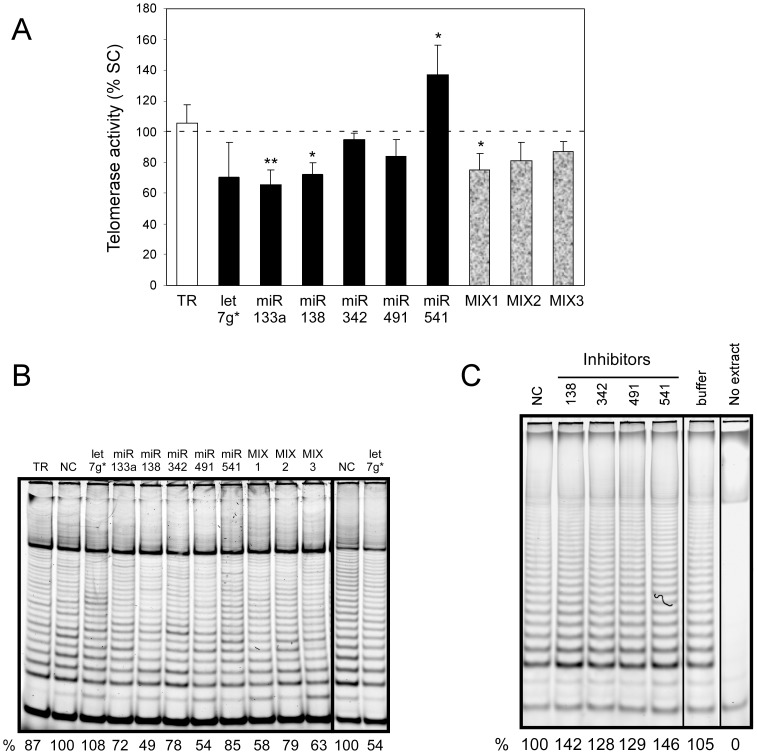
Multiple miRNAs decrease telomerase activity. (A) The cells were transfected by miRNA mimics or transfection reagent alone (TR). The mixtures of miRNAs as well as scrambled control (SC) were always at a concentration of 60 nM; MIX1 (miR-491, miR-541, and miR-342), MIX2 (let-7g*, miR-133a, and, miR-138), MIX3 (MIX1+MIX2). The telomerase activity in these cells determined 4 hours post transfection by a TRAP assay. Telomerase activity was evaluated by the intensity of the telomerase product ladder as measured using the Typhoon Trio scanner and Quantify software. Means and standard errors were calculated from 5 independent experiments. Downregulation of telomerase activity in HeLa cells transfected with miRNA mimic molecules is expressed as percent of telomerase activity in cells transfected with a scrambled control. Statistically significant differences relative to the negative control are expressed as in Figure 2. (B) Representative TRAP gel from the experiment. In this set let-7g* had slightly higher activity than control which resulted in bigger variance (see A). Therefore, we also provided let-7g* from another set together with appropriate control. (C) Jurkat cells were treated with miRNA inhibitors and cells were harvested for TRAP 48 hours after electroporation. Telomerase activity was evaluated by the intensity of the telomerase product ladder as described above.

The level of hTERT mRNA is critical for the regulation of telomerase activity in human cells. Therefore, we evaluated whether these miRNAs had the ability to inhibit telomerase activity in the HeLa adenocarcinoma cell line. miRNA precursors (60 nM of final concentration) were transfected into cells and telomerase activity was determined 4 hours after transfection using the telomeric repeat amplification protocol (Figure 3). We selected this time point to enhance the probability that the effect of miRNA on telomerase activity analyzed will represent a direct effect on its 3′UTR. hTERT mRNA is cell cycle regulated with the half-life 2–3 hours, therefore, there is sufficient mRNA turnover to detect the effect of miRNA treatment [40]. Telomerase activity was compared to the activity in cells transfected with control scrambled miRNA and transfection reagent alone. Most miRNAs decreased telomerase activity in the range of 10 to 30%. miRNAs miR-133a, miR-138, and miR-491, which specifically inhibited reporter activity, also directly inhibit telomerase activity in cells a few hours after treatment. miRNAs let-7g* and miR-491 also repeatedly reduced telomerase activity even though this effect was not statistically significant. Taken together, these results indicate that the regulatory mechanism of telomerase activity by these miRNA is directly through their binding to the 3′UTR of hTERT.

miRNAs regulating telomerase activity are expressed together in many normal tissues and their levels are significantly reduced in ovary and liver tumors suggesting that they may play role in the activation telomerase in these tissues [41]. To determine whether different miRNAs cooperate in the inhibition of telomerase activity, miRNAs were transfected in three different combinations into HeLa cells (Figure 3). The first combination contained three miRNAs (miR-491, miR-541, and miR-342) that have predicted binding sites in both the 3′UTR of hTERT and in the 3′UTRs of the TCF7, MSI1 and PAX5 genes (Figure 1) (MIX1). The second group contained three miRNAs that are conserved among Bilateria or vertebrates (let-7g*, miR-133a and miR-138) (MIX2). Finally, all six miRNAs were used in one transfection (MIX3). The final concentration of all mixes was 60 nM (i. e. the concentration of each miRNAs was actually lower than when transfected alone: 20 or 8.5 nM, respectively). Interestingly, the combination of miR-491, miR-541, and miR-342 (MIX1) inhibited telomerase activity more efficiently than any of these miRNAs alone. The reason for this effect may reflects the location of the miRNA binding sites in the 3′UTR of hTERT. miRNAs in MIX1 have binding sites close each to other in the middle of the 3′UTR and also have a relatively modest effect on telomerase activity as single miRNAs. Therefore, this can result in a greater potential for cooperation than for miRNAs in MIX2. The binding sites in the miRNA present in MIX2 are scattered over the entire length of the 3′UTR and individual miRNAs downregulate telomerase activity more efficiently than those in MIX1. The miRNAs in MIX3 are in two-times lower concentration than in MIX1 and MIX2. These results demonstrate that some of these miRNAs cooperate in the downregulation of telomerase activity by targeting the 3′UTR of hTERT.

To address the significance of regulation of telomerase activity by endogenous miRNAs we electroporated inhibitors of miR-138, miR-342, miR-491, and miR-541 into Jurkat cells in which these miRNAs are expressed [42]. Cells were harvested for TRAP analysis after 48 hours. All four inhibitors of miRNA increased the level of telomerase activity by 25–40%. These results suggest that endogenous miR-138, miR-342, miR-491, and miR-541 inhibit telomerase activity and their downregulation during tumorigenesis can results in activation of telomerase.

### miRNAs Participate in the Coordinated Regulation of Telomerase and TCF7, MSI1 and PAX5 Genes

**Figure 4:**
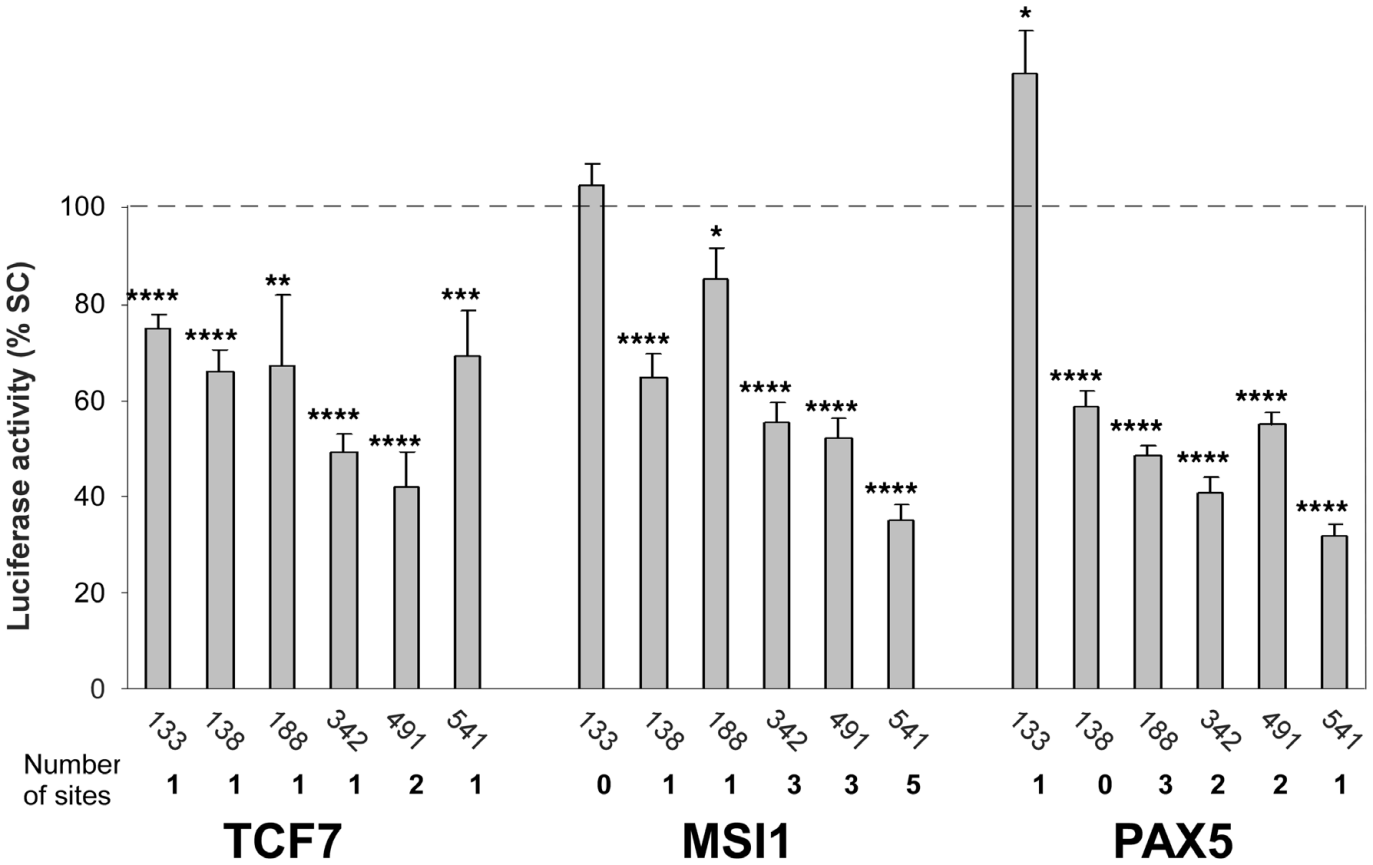
Downregulation of luciferase activity of the TCF7, MSI1, and PAX5 3′UTR reporters. The results are expressed as percent of reporter activity in cells transfected with a scrambled control (SC). Differences between the luciferase activity in SC and transfection agent treated cells were not statistically significant, demonstrating that transfection of miRNA did not induce a nonspecific effect (data not shown). Means and standard errors were calculated from 4 independent experiments. Statistically significant differences relative to the SC control are indicated as in Figure 2. Number of miRNA sites in respective 3′UTR is displayed. let-7g* has no predicted binding sites in the 3′UTRs of these three genes.

Six of the miRNAs which specifically inhibited the TERT 3′UTR-driven reporter activity also had predicted binding sites in the 3′UTRs of two or all three of the Wnt pathway regulating genes (TCF7, MSI1 and PAX5) analyzed (Figure 1b). To determine whether these miRNAs indeed regulate these genes through their 3′UTR we performed luciferase reporter assays (Figure 4). miR-133a, miR-138, miR-188, miR-342, miR-491, and miR-541 were co-transfected into HeLa cells with a reporter plasmid containing the appropriate 3′UTR and their ability to inhibit reporter activity was analyzed as described for the hTERT 3′UTR. Luciferase activity of the TCF7 reporter construct was inhibited in cells transfected with precursors of all tested miRNAs relative to cells transfected with the negative controls. The MSI1 reporter activities were also inhibited by all the miRNAs except miR-133a which has no predicted binding site in the MSI1 3′UTR. All miRNAs inhibited reporter activity in assays with the PAX5 3′UTR reporter including miR-138 (site not predicted by TargetScanHuman but several binding sites were identified by PITA and RNAhybrid programs) [43], [44]. Overall, miR-342, miR-491, and miR-541 demonstrated the greatest effect on all three reporters which is consistent since the 3′UTR of these genes have multiple predicted binding sites for the miRNAs examined. Though we did not perform assays with reporter where these predicted sites are mutagenized, our results demonstrated that several miRNAs which functionally interact with the 3′UTR of hTERT and inhibit telomerase activity are also likely involved in the regulation of genes involved in the Wnt signaling.

**Figure 5:**
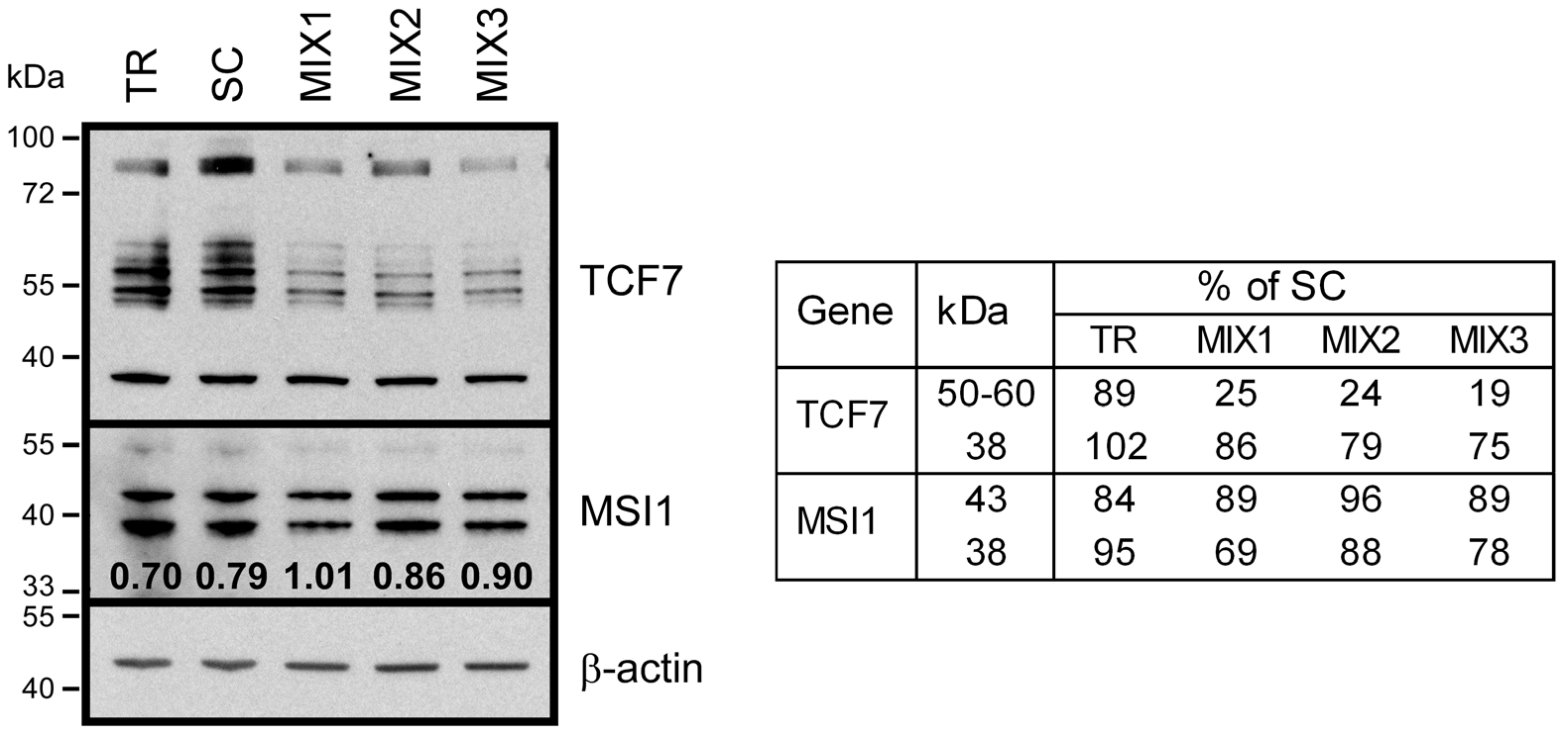
miRNAs targeting TERT regulate genes in the Wnt pathway. Western blot analysis of endogenously expressed TCF7 (38–80 kDa) and MSI1 (39 kDa and 43 kDa) proteins in DLD-1 cells transfected with scrambled control (SC), and MIX1, MIX2, and MIX3 as described in Figure 3 or with transfection reagent alone (TR). The mixtures of miRNAs as well as SC were at a concentration of 60 nM. Single transfected miRNAs gave more modest differences than mixtures. Molecular weight markers are shown in the left margin. β-actin served as a loading control. The quantification of Western blot analysis (Table) is shown on the right side. The signals of TCF7 or MSI1 were normalized to β-actin and expressed as a percentage of SC control. The ratio of 43 to 38 kDa MSI1 forms is shown on the picture under MSI1 bands.

The experiments above demonstrated that the transfection of the miRNAs (miR-138, miR-188, miR-342, miR-491, miR-541) inhibit TCF7 and MSI1 3′UTR reporters, therefore, we analyzed their ability to alter the endogenous protein levels of these genes in DLD-1 cells (Figure 5). We used this colon carcinoma cell line rather than HeLa cells because the Wnt signaling pathway is activated and significant levels of TCF7 and MSI1 protein are expressed relative to HeLa cells [45], [46]. TCF7 antibodies detected multiple proteins ranging from 38 to 60 kDa plus a high molecular mass band that may not be specific. A molecular species of TCF7 of this size has not been described. The predicted molecular mass of TCF7 is 42 kDa, however, instead multiple proteins ranging from 25 kDa to 60 kDa are usually detected [47]. This is at least in part, the consequence of the complex alternative splicing of TCF7 with more than 29 protein-coding variants [48]–[50]. The MSI1 antibodies detected two proteins approximately 39 and 43 kDa. The predicted MSI1 molecular mass is 39 kDa, but the protein is usually detected as of a doublet of two isoforms [51]. PAX5 expression was not detected in these cells. When three mixtures of miRNAs previously described were transfected and cells harvested after 48 hours, the expression of the TCF7 50–60 kDa proteins were decreased by all three combinations of miRNAs (by 70 to 80% of control). The overall expression of MSI1 isoforms was lower and the ratio changed in transfected cells because the expression of the 38 kDa protein decreased more than the 43 kDa protein. TCF7 and MSI1 are the principal regulators of the Wnt pathway [35]–[37]. Collectively, these results suggest that the hTERT targeting miRNAs are also involved in the regulation of some genes of the Wnt signaling pathway.

### miRNAs Modulate Cell Proliferation

**Figure 6:**
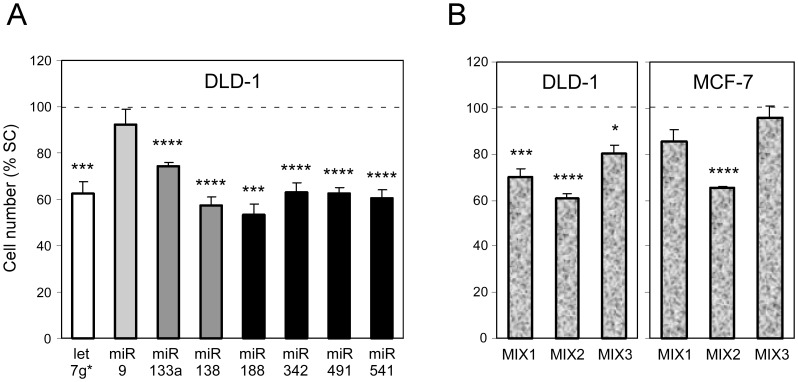
miRNAs targeting hTERT decrease cell proliferation in the DLD-1 and MCF-7 cell lines. The cells were transfected by miRNA mimics and cell numbers were determined 48 hours post-transfection. An increase in cell death was not detected (data not shown). Means and standard errors were calculated from 5 (single miRNAs) and 3 independent experiments (mixtures of miRNAs). Statistically significant differences relative to the negative control is expressed as in Figure 2. Differences between the SC and transfection agent treated cells were not statistically significant (data not shown). **(**a) Proliferation of DLD-1 cell transfected with miRNA mimic molecules are expressed as the percent of number of cells transfected with a scrambled control (SC). (b) Proliferation of DLD-1 and MCF-7 cells transfected with mixtures of miRNA mimics molecules expressed as a percent of the number of cells transfected with a scrambled control (SC).

A significant inhibition of HeLa cell proliferation following transfection of the hTERT regulating miRNAs was not observed (data not shown). HeLa cells have very low Wnt activity [52]. However, in cells in which the Wnt pathway is strongly activated such as the colon carcinoma cell lines DLD-1 and Caco-2, and the breast carcinoma cell line MCF-7 [45], [46], [53], cell proliferation was strongly inhibited after transfection with these miRNAs relative to control miRNAs. We quantified this effect by counting cells 48 hours after transfection (Figure 6a). The number of cells was significantly lower in cultures exposed to the miRNAs being analyzed than in cultures exposed to control miRNAs, with the exception of miR-9. We did not observed differences in number non adherent or dead cells. These results demonstrate that the selected miRNAs belong to a group of miRNAs which have the potential to inhibit the proliferation of some tumor cells.

We observed that hTERT specific miRNAs cooperate in their ability to reduce telomerase activity. In order to determine whether miRNAs may also cooperate to inhibit cell proliferation, DLD-1 and MCF-7 cell lines were treated with three combinations of miRNAs as described in Figure 3 with the difference that miR-188 was also a part of MIX1 and MIX3 (Figure 6b). In contrast to the effect on telomerase activity, hTERT specific miRNAs did not cooperate in the reduction of cell proliferation. When cells were treated with 60 nM of any of the miRNAs the level of inhibition of cell proliferation ranged from 25% to 45%. When a 60 nM mixture of the three miRNAs was used, the inhibition was 40% (MIX2), four miRNAs 15–30% (MIX1) and all seven miRNAs only 10–20% (MIX3) in both cell lines. These results suggest that the selected hTERT specific miRNAs fail to cooperate in inhibition of cell proliferation and their effect appears to be dependent on the concentration of the individual miRNAs.

## Discussion

The number of genes known to be regulated by miRNAs is exponentially increasing, however, only limited information is available about the direct regulation of hTERT by miRNAs [54]. miR-138 was demonstrated to directly bind its target site in the hTERT 3′UTR and repress hTERT protein expression [31]. Our report confirms this finding and demonstrates that at least five additional miRNAs (let-7g*, miR-133a, miR-342-5p, miR-491-5p, and miR-541-3p) directly regulate hTERT. Further, the transfection of four of these six miRNAs (let-7g*, miR-133a, miR-138, miR-491) also decreased telomerase activity. miR-9, which was also predicted by Target-scan to target the hTERT 3′UTR, failed to inhibit the hTERT reporter.

Our results indicate that the same set of miRNAs that interact with the hTERT 3′UTR also participate in the regulation of TCF7, MSI1, and PAX5 genes. TCF7 is one of the four essential transcription factors involved in the regulation of canonical Wnt signaling [35]. MSI1 (Musashi 1) has been shown to be both a target gene and an activator of Wnt and Notch signaling [36], [37]. This gene was shown previously to be regulated by miR-138 [55] and our results demonstrated that it is also under regulation of miR-188, miR-342, miR-491, and miR-541. PAX5 upregulates LEF1 (TCF7-related protein, regulator of Wnt signaling) and interacts directly with LEF1 in B cells [38], [39]. Finally, the observation that PAX5 is a transcriptional regulator of the hTERT gene further increases the complexity of this network [56]. The interplay of shared miRNA regulators between the Wnt pathway and telomerase is likely to have functional consequences. Previously, telomerase function has been linked with the activation of Wnt signaling pathway [25]. The hTERT protein was shown to bind to a chromatin-remodeling protein BRG1 to stabilize the TCF/β-catenin transcription complex on the promoters of Wnt-regulated genes and increase their rate of transcription. By employing the same miRNAs in the regulation of hTERT and genes in the Wnt pathway, a regulatory network could be established to control both telomerase activity and cell proliferation. This network would be important in cells where telomerase and Wnt pathways are both involved such as in stem cells or several tumor types [57], [58].

The inhibition of the luciferase reporter and inhibition of telomerase activity four hours after miRNA treatment suggest that interactions of miRNAs with the hTERT 3′UTR directly mediate these effects through the regulation of hTERT. Some of these miRNAs cooperate, even at low concentration, in the inhibition of telomerase activity as described previously for inhibition of other genes [59], [60]. The inhibition of hTERT itself is able to decrease cell proliferation, however, the effect of these miRNAs on proliferation was pronounced only in cells with a highly activated Wnt pathway, suggesting that the inhibition of Wnt target genes by these miRNAs was responsible for most of this effect. Accordingly, the overexpression of MSI1, which is also a target of miR-138, abrogated the inhibitory effect of miR-138 on cell proliferation [55]. These results suggest that the coordinated expression of multiple miRNAs targeting one gene might cooperate in inhibition even at low levels. In contrast, some complex biological processes may be dependent on reaching a certain threshold concentration of a particular miRNA to produce a regulatory effect. Furthermore, even when binding sites of several miRNAs are present within 3′UTR of one gene, miRNAs may not always be able to cooperate because their relative location and stoichiometry will not allow it.

While miRNAs can function both as oncogenes and anti-oncogenes, miRNAs that target the hTERT 3′UTR inhibit tumorigenesis and consequently are commonly downregulated in many types of cancer. miR-138 was found to be downregulated in anaplastic T cell (ATC) leukemia and in aggressive variants of papillary thyroid carcinomas (PTC), in oral and head and neck squamous cell carcinomas (OSCC and HNSCC) as well as in lung, hepatic and gastric tumors [15], [31], [61]–[65]. This miRNA was shown to decrease cell motility, inhibit the DNA-damage response, decrease cell proliferation, and the ability of the tumor cells to form metastases [61], [62], [64], [66]. There is no information on the biological function of the minor strand, let-7g*, that targets hTERT. However, the let-7g strand, which is expressed as a part of the same pre-miRNA precursor, inhibits cell migration of hepatocellular carcinoma cells and contributes to the development of the metastatic state of breast cancer [67], [68]. miR-342, is downregulated in tamoxifen-resistant breast tumors and in lung carcinomas, inhibits proliferation, invasiveness of colorectal cancer cells, induces apoptosis in cutaneous lymphoma and differentiation of leukemic cells [69]–[73]. miR-491-5p induces apoptosis in colorectal cancer cells by binding BCL2L1 (Bcl-X_L_) and inhibits cellular invasion of glioma cells [74], [75]. miR-541 is expressed at lower levels in recurring adrenal pheochromocytomas relative to benign tumors [76]. Both miR-491-5p and miR-541 also inhibit androgen-induced proliferation of prostate cancer cells [77]. Finally, miR-133a/b belongs to the group of miRNAs most consistently downregulated in the wide variety of solid tumors [78]–[80]. In contrast, miR-9, which has no effect in the hTERT 3′UTR reporter assay, is principally known as a tumor promoting miRNA that is upregulated by c-Myc and N-Myc and promotes the metastatic behavior of cancer cells [81]. In conclusion, all the miRNAs identified as hTERT regulators have been classified as tumor suppressors, which is consistent with their ability to inhibit proliferation of tumor cell lines. There is also a possibility that longer exposure of transformed cells to these miRNAs may results in apoptosis.

Our results suggest that more miRNAs are likely involved in the hTERT regulatory network. It is likely that there are additional miRNAs that co-regulate hTERT with other genes through binding to their 3′UTR because we did not analyze the less conserved miRNAs and TargetScan5.2 did not consider all human miRNAs known at that time. Additionally, it is likely that the rules for predicting the sequence of the binding sites are too strict and a shorter precise match in the seed sequence than currently considered may be sufficient for miRNA binding [82]. This is consistent with several reports in the last two years that concluded that multiple miRNAs interact with the 3′UTR of a single gene. For example, 28 miRNAs bind the 1.5 kb 3′UTR of cyclin-dependent kinase inhibitor 1A (CDKN1A) and 8 miRNAs target 0.43 kb 3′UTR of mouse Myc [83], [84]. The functional interaction of a single gene with multiple miRNAs suggests a complex regulation by miRNAs similar to a transcriptional regulatory network. Both the number of human miRNAs and their targets are sufficient to provide for such a network. The current estimates of miRNA targets include most mammalian genes and the recent miRNA database (miRBase 18) lists 1921 mature miRNAs encoded by 1527 hairpin genes in *Homo sapiens*, which is very similar to the number of sequence-specific transcription factors [34], [85], [86]. These data strongly suggest that miRNA regulation has evolved as a complex network which interfaces with the entire transcriptome.

## Conclusion

In conclusion, multiple miRNAs regulate telomerase activity, proliferation, TCF7, MSI1 and PAX5 genes. Thus, small non-coding miRNAs may be involved in a coordinated regulatory network involving the transcriptional regulation of telomerase and several genes of the Wnt pathway.

## Methods

### Recombinant DNA Construction

The entire 3′UTR sequence of the hTERT gene (GenBank: NM_198253.2, nt 3458-4018) was PCR amplified from human cDNA and cloned into the XbaI-XhoI sites of the pcDNA3.1dsRluc *Renilla* luciferase reporter vector [87]. 3′UTR reporter constructs with mutated miRNA target sites were constructed with the QuikChange Site-Directed Mutagenesis Kit (Agilent Technologies, Santa Clara, CA) (Figure S1). The LightSwitch 3′UTR GoClone reporters of TCF7, MSI1, and PAX5 were obtained from SwitchGear Genomics (Menlo Park, CA).

### Cell Lines and Tissue Culture

The Jurkat, HeLa, DLD-1, MCF-7 and Caco-2 cells were the gifts from P. Tucker, C. Sullivan, and J. Dudley (University of Texas, Austin). DLD-1 and Caco-2 colon carcinoma cell lines were described previously [88], [89]. The cell lines were cultured in Dulbecco’s modified Eagle’s medium supplemented with 10% fetal calf serum (Atlanta Biologicals, Norcross, GA). HeLa, DLD-1, Caco-2, or MCF-7 cells were plated in 6-well (8×10^4^ cells per well) or 12-well plates (4×10^4^ cells per well) and transfected after 24 hours using siPORT amine reagent (Ambion, Austin, TX) or Metafectene SI (Biontex-USA, San Diego, CA) with miRNA mimics (Ambion) at a final concentration of 60 nM (Table S1). All cells (including floating cells) were harvested after 48 hours, counted using a hemocytometer, and cell extracts were prepared for Western blot analysis. Trypan blue staining was used to evaluate the number of dead cells. For TRAP analysis cells were harvested 4 hours after transfection. miRNA inhibitors (Ambion) were electroporated into Jurkat cells under 200 V, 1 µF in siPORT electroporation buffer (Ambion) using Gene Pulser II electroporation apparatus (BioRad).

### Reporter Assays

The HeLa cells were transfected in 12-well plates with the *Renilla* luciferase-based hTERT 3′UTR reporter constructs using FuGENE HD transfection reagent (Promega, Madison, WI). Transfection efficiency was controlled by co-transfection with a firefly luciferase-based control reporter pCI-firefly [90] and the pEGFP-N1 fluorescent reporter (Clontech Laboratories, Mountain View, CA). After 24 hours the cells were super-transfected with miRNA mimics at 60 nM final concentration using siPORT amine reagent (Ambion). Cells were harvested 72 hours after reporter transfection and analyzed using a Dual-Luciferase Reporter Assay System (Promega). The reporter assay employing constructs with TCF7, MSI1, and PAX5 3′UTR were performed similarly with the following modifications: the HeLa cells were co-transfected in 24-well plates with the *Renilla* luciferase-based UTR reporter constructs, pCI-firefly, and pEGFP-N1 reporters, and 60 nM miRNA mimics using Metafectene Easy transfection reagent (Biontex-USA). Cells were harvested 48 hours after transfection and analyzed using a Dual-Luciferase Reporter Assay System (Promega). The *Renilla* luciferase signal of each sample was normalized for differences in transfection efficiency using the activity of co-transfected pCI-firefly reporter as control.

### Western Blot Analysis

TCF7 was detected with the mixture of antibodies 1D2 and 2E9 (Novus Biologicals, Littleton, CO). The MSI1 protein was detected by monoclonal antibody (EP1302) (Abcam, Cambridge, MA). Human actin was stained by monoclonal antibody AC-15 (ab6276) (Abcam). The cells were lysed in buffers supplemented with a set of inhibitors which included: 2× concentrated complete protease inhibitor cocktail (Roche Diagnostics, Indianopolis, IN), 2 mM phenylmethylsulfonyl fluoride, and aprotinin (Sigma-Aldrich, St. Louis, MO). Prestained molecular weight markers EZ-RUN from Thermo Fisher Scientific (Walthman, MA) were employed to determine molecular weights. Proteins were separated on NuPAGE 4–12% Bis-Tris gels, electroblotted to Invitrolon PVDF membrane (Invitrogen, Carlsbad, CA) and visualized with SuperSignal West Dura Chemiluminescent Substrate with (MSI1) or without (TCF7) SuperSignal Western blot enhancer (Thermo Fisher Scientific).

### Telomeric Repeat Amplification Protocol (TRAP)

The level of telomerase activity was evaluated using the TRAP assay [91]. Whole cell extracts were prepared with CHAPS buffer [18]. Equivalent amounts of extracts (based on total protein content) were incubated with the unlabeled TS primer in dNTP-containing TRAP buffer for 45 min at 37°C. The reaction was stopped at 94°C for 2 min and aliquots of synthesis were PCR amplified with using KAPA2G Robust DNA polymerase (Kapa Biosystems, Woburn, MA), Cy5-labeled-TS primer, ACX primer and NT/TSNT control primers. The TRAP PCR products were separated on acrylamide gels and images were captured using a Typhoon Trio imager (GE Healthcare, Waukesha, WI). The images were quantified using Quantity One software (BioRad, Philadelphia, PA).

## Supporting Information

Figure S1 **hTERT 3′UTR reporter with miRNA binding sites and mutagenized nucleotides.**
(PDF)

Methods S1 **Prediction of genes that share 3′UTR miRNA target sites with hTERT 3′UTR.**
(PDF)

Table S1 **miRNAs used in the study.**
(PDF)
