# Supplementary material for: Multiple Tumor Suppressor microRNAs Regulate Telomerase and TCF7, an Important Transcriptional Regulator of the Wnt Pathway
Source: PLoS One. 2014 Feb 14;9(2):e86990. doi: 10.1371/journal.pone.0086990 (PMC3925088; doi:10.1371/journal.pone.0086990)
Supplement: Figure S1 — hTERT 3′UTR reporter with miRNA binding sites and mutagenized nucleotides. (PDF) [file pone.0086990.s001.pdf]

Figure S1. hTERT 3'UTR reporter sequence (shown as RNA transcript) with miRNA binding sites and mutagenized nucleotides indicated. The human 3'UTR (561 nt) was cloned into XhoI-XbaI (green highlights) of the reporter vector. The miRNA seed sequences and complementary binding sequences in hTERT 3' UTR are indicated using the same color schema as in the Tab. S1. Three consequent nucleotides which were mutagenized on the DNA template in each miRNA binding site are underlined and nucleotides which replaced them are shown below. The specific miRNAs and their binding are indicated.

```
                        GCCGGACUAAGUGUUGUGGUCGA  <hsa-miR-138-5p
                        | | | ||     :||||||||
CUCGAGUGGCCACCCGCCCACAGCCAGGCCGAGAGCAGACACCAGCAGCCCUGUCACGCCGGGCUCUACGUCCCAGGGAGGGAGGGGCGGCCCACACCCAGGCCCGCACCGCUGGGAGUC        120 (114)
                                          ACU

                                                                                          hsa-miR-491-5p>  GGAGUACCUUCCC
                                                                                                           |: |
UGAGGCCUGAGUGAGUGUUUGGCCGAGGCCUGCAUGUCCGGCUGAAGGCUGAGUGUCCGGCUGAGGCCUGAGCGAGUGUCCAGCCAAGGGCUGAGUGUCCAGCACACCUGCCGUCUUCAC        240 (234)

             hsa-miR-342-5p                              hsa-miR-491-5p
AAGGGGUGA  AGUUAGUGUCUAUCGUGGGGA                      GGAGUACCUUCCCAAGGGGUGA
||||||||    :::   : |:   ||||||                          |  |       |||||||
UUCCCCACAGGCUGGCGCUCGGCUCCACCCCAGGGCCAGCUUUUCCUCACCAGGAGCCCGGCUUCCACUCCCCACAUAGGAAUAGUCCAUCCCCAGAUUCGCCAUUGUUCACCCCUCGCC        360 (354)
   AAA                     AAA                                        AAA

                                                                GUCGACCAACUUCCCCUGGUUU   <hsa-miR-133a
                                                                | |    || :: | |||||||
       UCAGGUCUAAGACACGGGUGGU  <hsa-miR-541-3p                   AGUAUGUCGAUCUAUUGGUUUCU  <hsa-miR-9-5p
         |:::  |||    |||||||                                    | : |   : : :|:|||||||:
CUGCCCUCCUUUGCCUUCCACCCCCACCAUCCAGGUGGAGACCCUGAGAAGGACCCUGGGAGCUCUGGGAAUUUGGAGUGACCAAAGGUGUGCCCUGUACACAGGCGAGGACCCUGCACC        480 (474)
                         CAA                                                     AAC             CCA
                                                                                  ||| |  ||| ||||||||
                                                                hsa-let-7g-3p>   CGUUCCGUCACCGGACAUGUC

                          ACGUUUGGGACGUACACCCUC  <hsa-miR-188-3p
                          || :::       ||||||||
UGGAUGGGGGUCCCUGUGGGUCAAAUUGGGGGGAGGUGCUGUGGGAGUAAAAUACUGAAUAUAUGAGUUUUUCAGUUUUGAAAAAAAUCUAGA                                   573 (561)
                                          UUU
```
