# Supplementary material for: Multiple Tumor Suppressor microRNAs Regulate Telomerase and TCF7, an Important Transcriptional Regulator of the Wnt Pathway
Source: PLoS One. 2014 Feb 14;9(2):e86990. doi: 10.1371/journal.pone.0086990 (PMC3925088; doi:10.1371/journal.pone.0086990)
Supplement: Methods S1 — Prediction of genes that share 3′UTR miRNA target sites with hTERT 3′UTR. (PDF) [file pone.0086990.s002.pdf]

Methods S1. Prediction of genes that share 3'UTR miRNA target sites with hTERT 3'UTR

TargetScanHuman 5.2 predicts in hTERT 3'UTR 47 binding sites (as defined in miRBase database, Release 18) for the miRNAs with 44 distinct seed sequences. From this pool, 24 seed sequences were selected that are present in miRNAs that belong to the highly conserved families (miR-9, miR-129-3p, miR-133ab, miR-138, miR-188-3p, miR-299/299-3p, miR-320/320abcd, miR-328, miR-337-3p, miR-342-5p, miR-346, miR-483/483-3p, miR-491/491-5p, miR-510, miR-512-5p, miR-532-3p, miR-541/654-5p, miR-542/542-3p, miR-632, miR-668, miR-671-5p, miR-767-3p, miR-1248, miR-1291). These sequences were used to search for other genes that contain sites complementary to these seed sequences in their 3'UTR using the algorithms built in the TargetScan, without employing evolutionary conservation filter. Twenty four gene pools identified by TargetScan were then searched for the genes with the highest number and density of sites complementary to these seed sequences using common functions built in the Microsoft Excel spreadsheet program. This analysis identified a group of 348 genes that share nine or more predicted sites with the hTERT 3'UTR and had the high density of shared predicted binding sites (at least one site per 188 bp). The genes with 3'UTR sites complementary to seed sequences found in hTERT 3'UTR were sorted into groups based on the number of potential miRNA binding sites shared between the respective gene and the hTERT 3'UTR. This analysis identified a group of approximately 350 genes that share nine or more predicted sites with the hTERT 3'UTR and have high density of shared predicted binding sites. The function of the most of these genes is unknown. We focused to genes which are known regulators of specific transcription of genes involved in signaling pathways. In this way, we have selected for initial screening six genes encoding different DNA- or RNA-binding proteins that function in various pathways (KLF15, MEOX1, MSI1, PAX5, TCF7, and ZNF362). One of these factors, TCF7, was known

regulator of Wnt signaling, the pathway that was previously linked to the function of telomerase. Therefore we have added one more Wnt pathway gene, LRP4, found in 350 selected genes, to the initial screen. These seven genes were evaluated to determine whether their expression could be affected by the overexpression of the hTERT 3'UTR in the cells, anticipating that it would act as a sponge and decrease the pool of miRNAs. cDNAs were prepared from Saos-2 cells overexpressing the hTERT 3'UTR or an empty vector control and the levels of mRNA of the seven selected genes were determined by RT-PCR. These cells were selected for the screen because they express relatively low level of endogenous hTERT so we anticipated the significant sponge effect of exogenous hTERT 3'UTR. The expression of three genes, TCF7, MSI1, and PAX5, which were not detected in control Saos-2 cells, appeared in cells overexpressing the hTERT 3'UTR (data not shown). These three genes were selected for further analysis.
