# Supplementary material for: Multiple Tumor Suppressor microRNAs Regulate Telomerase and TCF7, an Important Transcriptional Regulator of the Wnt Pathway
Source: PLoS One. 2014 Feb 14;9(2):e86990. doi: 10.1371/journal.pone.0086990 (PMC3925088; doi:10.1371/journal.pone.0086990)
Supplement: Table S1 — miRNAs used in the study. (PDF) [file pone.0086990.s003.pdf]

Table S1. miRNAs used in this study

Name[a]	miRBase accession #[b]	miRNA[c]
hsa-miR-138-5p	MIMAT0000430	A**GCUGGU**GUUGUGAAUCAGGCCG
hsa-miR-491-5p	MIMAT0002807	A**GUGGGG**AACCCUUCCAUGAGG
hsa-miR-342-5p	MIMAT0004694	A**GGGGUG**CUAUCUGUGAUUGA
hsa-miR-541-3p	MIMAT0004920	U**GGUGGG**CACAGAAUCUGGACU
hsa-miR-9-5p	MIMAT0000441	U**CUUUGG**UUAUCUAGCUGUAUGA
hsa-miR-133a	MIMAT0000427	U**UUGGUC**CCCUUCAACCAGCUG
hsa-let-7g-3p	MIMAT0004584	C**UGUACA**GGCCACUGCCUUGC
hsa-miR-188-3p	MIMAT0004613	C**UCCCAC**AUGCAGGGUUUGCA

[a] miRNA ID

[b] miRBase accession number

[c] Sequence of miRNA with the seed sequence (nt 2-7) shown in colored bold letter.
